# Molecular Mechanisms of Oxytocin Signaling at the Synaptic Connection

**DOI:** 10.1155/2018/4864107

**Published:** 2018-07-02

**Authors:** Jan Bakos, Annamaria Srancikova, Tomas Havranek, Zuzana Bacova

**Affiliations:** ^1^Institute of Experimental Endocrinology, Biomedical Research Center, Slovak Academy of Sciences, Bratislava, Slovakia; ^2^Institute of Physiology, Faculty of Medicine, Comenius University, Bratislava, Slovakia

## Abstract

Aberrant regulation of oxytocin signaling is associated with the etiology of neurodevelopmental disorders. Synaptic dysfunctions in neurodevelopmental disorders are becoming increasingly known, and their pathogenic mechanisms could be a target of potential therapeutic intervention. Therefore, it is important to pay attention to the role of oxytocin and its receptor in synapse structure, function, and neuron connectivity. An early alteration in oxytocin signaling may disturb neuronal maturation and may have short-term and long-term pathological consequences. At the molecular level, neurodevelopmental disorders include alterations in cytoskeletal rearrangement and neuritogenesis resulting in a diversity of synaptopathies. The presence of oxytocin receptors in the presynaptic and postsynaptic membranes and the direct effects of oxytocin on neuronal excitability by regulating the activity of ion channels in the cell membrane implicate that alterations in oxytocin signaling could be involved in synaptopathies. The ability of oxytocin to modulate neurogenesis, synaptic plasticity, and certain parameters of cytoskeletal arrangement is discussed in the present review.

## 1. Introduction

Although it has been known for a long time that oxytocin exerts modulatory effects on synaptic activity, recent studies have produced many important findings related to the neuron and glial cell structure, development, and functionality. In a broader sense, oxytocin is one of the most studied molecules in the context of brain development and social behavior. Oxytocin acts on oxytocin and partially on vasopressin receptors; therefore, regardless of focus on the oxytocin system, it is important to keep in mind the significant receptor cross-reactivity for their endogenous ligands [1, 2]. Functional oxytocin receptors have been recently discovered at neural progenitor cells which implies that they could be associated with cell fate selection [3]. Furthermore, oxytocin plays a role in the early development of neurons and participates in synapse formation [4]. Oxytocin-producing cells appear during the early phase of the brain development [5], and their maturation, particularly their ability to produce oxytocin, may influence the formation of neural circuits [6].

With regard to the defects in oxytocin signaling, there is a whole spectrum of neurodevelopmental disorders frequently associated with them, which include autism; therefore, it is increasingly important to pay attention to the role of oxytocin and its receptor in synapse structure, function, and neuron connectivity. An early alteration in oxytocin signaling may disturb neuronal maturation and may have short-term and long-term pathological consequences [7]. At the cell and molecular level, autism is a heterogeneous disorder, and its pathology includes alterations in cytoskeletal rearrangement, neuritogenesis, and elongation of axons and dendrites resulting in a diversity of synaptopathies [8]. In particular, impaired synapse formation results in disrupted neuronal connectivity and circuit stabilization which in turn can explain the pathogenesis of the disease [9]. Given the growing prevalence of autism, identification of risk factors and therapeutic interventions is a key factor of the relevant objectives of the current neurobiology research. The ability of oxytocin to modulate neurogenesis, synaptic plasticity, and certain parameters of cytoskeletal arrangement is discussed in the following review.

## 2. Oxytocin Receptors in Neurons

It is well known that receptors for neuropeptides are found heterogeneously distributed throughout the brain and may be expressed on cell bodies, dendrites, and axon terminals [10]. Oxytocin receptors are distributed in all brain regions, and oxytocin-producing neurons project from the hypothalamus to various brain areas including the limbic regions and brain cortex [11]. Oxytocin receptors are among the group of G protein-coupled receptors. Different intracellular pathways are activated by oxytocin according to the specific G proteins that they activate [2]. In neurons, an oxytocin receptor is coupled to G_*α*i_ and G_*α*q_, with protein kinase C/phospholipase C*β* as downstream effectors, rather than cAMP [12].

Modern techniques allow for specifically tracing and optogenetically stimulating oxytocin receptor-expressing neurons in different brain regions in order to observe the effects on thirst-related neural circuits or social behavior-related circuits [13, 14]. A recent study has discovered that oxytocin promotes the survival and maturation of newborn neurons in the hippocampus via its receptor [15]. Stimulation of neurogenesis in the hippocampus by oxytocin has been observed in previous studies [16]. This finding could be extended to other brain areas as one study has already proved neurogenesis in oxytocin-containing neurons in the hypothalamus [17]. Moreover, oxytocin may affect expression of neurotrophic factors such as the brain-derived neurotrophic factor (BDNF) and the nerve growth factor (NGF), which contribute to neural plasticity [18]. In the following study, we have observed that oxytocin affects the expression of neuron and glial markers in the brain [19]. Therefore, it can be suggested that the oxytocin system is involved in the regulation of development of neuronal precursor cells in the brain. This is in line with the recent study suggesting the role of oxytocin in neural progenitors [3]. The authors concluded that exposure to oxytocin increases generation of neurons and decreases production of oligodendrocytes and astrocytes [3]. It is generally accepted that oxytocin receptors are localized in neurons and glial cells as well; however, the majority of studies are focused on the effects of oxytocin on excitability of the neuron cell membrane [20].

Neurons constantly undergo structural and functional alterations as part of a process called synaptic plasticity; therefore, there is no doubt that oxytocin could affect synaptic activity at many levels of regulation. A recent study has demonstrated that the oxytocin receptor mRNA could be detected during mouse embryonal development in both sexes, although females appear to have more of the oxytocin receptor mRNA than males [21]. These differences between males and females may relate to hormonal differences, which alter oxytocin signaling. These findings are especially important in the context of development of neural circuits and their potential alterations. It is necessary to emphasize that some studies suggest that oxytocin inhibits fetal neurons resulting in the switch of GABAergic neurons from excitatory to inhibitory [22, 23]. Oxytocin receptors in neurons could represent a critical factor in organizing neural circuitry and the development of social behavior.

## 3. The Role of Oxytocin in Neural Circuits

Balance between excitation and inhibition is an important feature of neuronal circuits. Oxytocin can directly increase neuronal excitability by regulating the activity of ion channels in the membrane and thus modulating synaptic transmission [24]. However, the extent of oxytocin action depends on the brain region, type of neurons, and their initial membrane excitability. Over the past decade, both *in vivo* and *in vitro* studies have made the progress from investigating electrical spiking activity of the oxytocin neurons ranging from the hypothalamus, the hippocampus, and the amygdala to the spinal cord [25–27]. Originally, even though electrical properties of oxytocin-producing neurons have been mostly related to parturition and/or lactation, nowadays it is clear that the wide spectrum of physiological functions including analgesia and regulation of behavior is associated with oxytocin. Indeed, oxytocin actions on synaptic transmission in the adult brain are often specific in the context of social communication [28]. Oxytocin induces excitation of neurons in the amygdala, [29] and it could be suggested that together with the effects on hippocampal formation, oxytocin is involved in information processing in limbic circuits and plays a role in memory and learning regulation [30]. Furthermore, many studies have implicated oxytocin with anxiolytic effects in the limbic areas and prefrontal cortex [31]. Nevertheless, in contrast to previous studies, it has been found that oxytocin produces antinociception in part by reducing excitatory neurotransmitter release [32]. These authors explain their findings by oxytocin modulation of high voltage-gated calcium channels, primarily of the N-type, to reduce presynaptic glutamate release [33]. Many studies claim that oxytocin released from the hypothalamus is distributed through oxytocin-containing fibers to the spinal cord and has stress-inducing analgesic effects [34, 35]. In a broader sense, neuropeptide modulation of GABA and glutamate synaptic actions are complex and require knowledge of the cell type, brain region, composition of synaptic membrane, and coupling of receptors to ion channels. In the case of oxytocin, it is important to consider the effects of oxytocin on both oxytocin and vasopressin receptors. It might be especially important to know how balanced these receptors are in the synaptic cleft.

## 4. Presynaptic Modulation by Oxytocin

**Figure 1:**
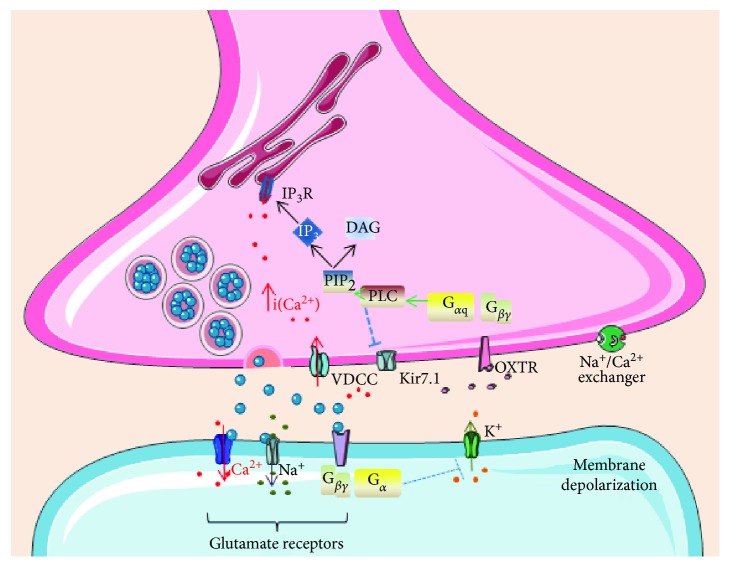
Presynaptic G protein-coupled oxytocin receptors modulate membrane polarity. OXTR: oxytocin receptor; VDCC: voltage-dependent calcium channels; PIP_2_: phosphatidylinositol biphosphate; IP_3_: inositol 1,4,5-trisphosphate; DAG: diacylglycerol.

Presynaptic oxytocin receptors were described in the 1980s by Audigier and Barberis [36], and at that time, there were already speculations on how oxytocin could affect neurotransmission [37]. These authors [36] isolated synaptic plasma membranes and proved that they contain binding sites for oxytocin and vasopressin. Since that time, the presence and functionality of oxytocin receptors at the synapse have been repeatedly proved in many areas of the central and peripheral nervous systems. Activation of the oxytocin receptor on the presynaptic membrane, resulting in an increase in intracellular calcium concentration, may increase the secretion of the neurotransmitter into the synaptic cleft. Oxytocin stimulates the increase of calcium through two major mechanisms (Figure 1). First is through G_*α*q_-mediated activation of the phospholipase C, which by the action of inositol-triphosphate binding on inositol 1,4,5-trisphosphate receptor (IP_3_R) induces release of calcium from intracellular sources [38]. Second is through inhibition of the potassium Kir7.1 channels, which induces plasma membrane depolarization and calcium entry through the voltage-dependent calcium channel [39, 40]. Another mechanism on how oxytocin may induce membrane depolarization on presynaptic membranes is through activation of Na^+^/Ca^2+^ exchanger and the opening of a nonselective cation channel [41]. The activation of the Na^+^/Ca^2+^ exchanger by G protein-coupled receptors has been suggested in different hypothalamic systems [42, 43].

### 4.1. Excitatory Synapse

Modulation of excitatory synaptic transmission by the action of oxytocin on its receptors is complex and may differ in various parts of the brain. A recent study has demonstrated that the activation of presynaptic oxytocin receptors enhances the release of depolarization-evoked glutamate in the hippocampus [44]. Nevertheless, other brain regions are also the target of oxytocin effects. It has been demonstrated that the activation of oxytocin receptors facilitates glutamatergic synaptic transmission in the spinal cord [45]. These authors explained their findings in the way that oxytocin acts on presynaptic membrane of subpopulation of glutamatergic neurons and as a result of this action promotes release of the neurotransmitter. This release of glutamate results in excitation of GABAergic neurons and when connected to further neurons transmits inhibitory message on them. This complicated concept is used in current studies for explanation of the nociceptive effects of oxytocin. Nevertheless, it is likely that interneurons in the spinal cord may use oxytocin as a neurotransmitter. It also means that synaptic contacts with neurons in the second or third order could be under a different oxytocin modulation. Contradictory to the results that describe the enhancement of excitatory synaptic transmission, there are also suggestions that oxytocin works against glutamate release from the presynaptic membrane. It has been revealed that dendritically released oxytocin decreases evoked excitatory synaptic transmission by inhibiting glutamate release from the presynaptic terminals [46]. The authors of this study explain the effect of oxytocin by evidence of modulation of voltage-dependent calcium channels, mainly N-type and to a lesser extent P/Q-type channels, located on glutamatergic terminals. This concept suggests the existence of two-way communication between the presynaptic terminal and the postsynaptic synaptic membrane. Although in our study we did not distinguish between presynaptic/postsynaptic actions of oxytocin, we confirmed that oxytocin acts via a mechanism involving the N-type channels and P/Q-type channels inducing neurite outgrowth [47]. It is widely accepted that the presynaptic effect of oxytocin is dependent on extracellular calcium [48]. Furthermore, oxytocin receptors were found at the so-called putative excitatory synapses, both at presynaptic and postsynaptic terminals and at inhibitory synapses as well [49]. Taken together, the results of the available studies are not conclusive leaving a room for various interpretations. Therefore, more studies can bring more specific data on oxytocin action in excitatory synapse.

### 4.2. Inhibitory Synapse

**Figure 2:**
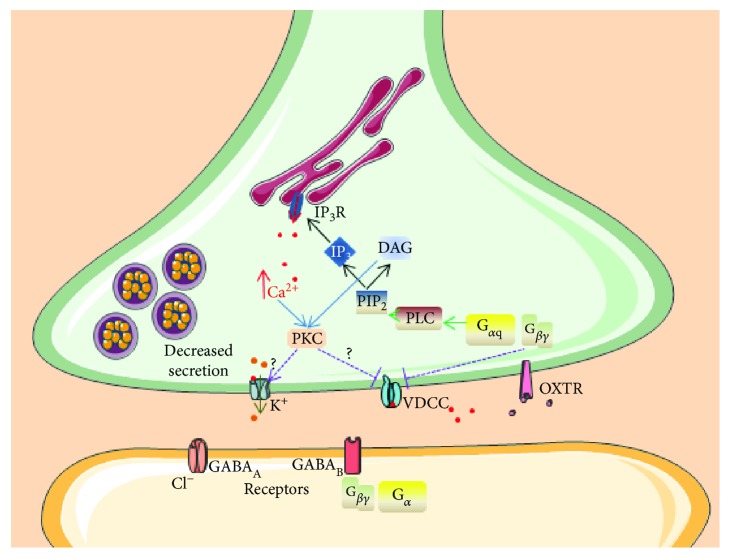
Presynaptic G protein-coupled oxytocin receptors modulate neurotransmitter release. OXTR: Oxytocin receptor; VDCC: voltage-dependent calcium channels; PIP_2_: phosphatidylinositol biphosphate; IP_3_: inositol 1,4,5-trisphosphate; DAG: diacylglycerol; GABA: *γ*-aminobutyric acid receptor.

The active role of oxytocin in inhibitory synapses could also be a matter of discussion. Some studies explain the role oxytocin in inhibitory terminals in the way that oxytocin acts via presynaptic activation of oxytocin receptors resulting in a decrease of GABAergic transmitter release [49]. In this study, the authors suggest that the predominant effect of oxytocin modulation is to reduce inhibitory transmission without directly affecting excitation. The pathways responsible for the decrease of GABA secretion (Figure 2) include the inhibition of voltage-dependent calcium channels by protein kinase C and/or calcium-dependent potassium channels [50]. This conclusion is supported by other studies in different brain areas: the auditory cortex, piriform cortex, hypothalamus, and hippocampus [51, 52]. Conversely, another study has suggested that oxytocin depresses spontaneously occurring GABA receptor-mediated inhibitory potential by acting presynaptically in the olfactory blub [53]. This is supported by the inhibitory effects of oxytocin on different sets of interneurons *in vitro* [54]. Taken together, the activity of oxytocin on its receptors at the presynaptic membrane depends on the neuron type and brain area and most likely also on the complex presynaptic inputs including astrocyte-originated transmission [55].

## 5. Postsynaptic Modulation by Oxytocin

Regulation of postsynaptic membrane potential represents a significant part of oxytocin's contribution to the modulation of synaptic function. Oxytocin can directly act via oxytocin receptors on postsynaptic neurons in order to alter the activity of neural circuits which regulate social behaviors [56, 57]. Yao et al. [56] discovered the presence of oxytocin receptors in a special subpopulation of neurons in the amygdala which express the steroid-converting enzyme aromatase. This finding has extended the knowledge of oxytocin receptor stimulation to specific female-evoked neural responses and behaviors in the male mouse. The effect of oxytocin on membrane excitability of rat dorsal root ganglion neurons has also been investigated [58]. The authors found that oxytocin significantly decreased the amplitude of the depolarization and number of action potentials induced by acid stimuli. In this context, the authors also stressed the role of vasopressin receptor V1a. Another recent study has demonstrated that oxytocin enhances the frequency of spontaneous inhibitory postsynaptic currents [59]. It is relevant to note that the authors also suggest that both V1a and oxytocin receptors play an important role. Another study has found that oxytocin induces an increase in intracellular calcium on the postsynaptic membrane, which consequently depresses inhibitory synaptic transmission [60]. Oxytocin affects the spontaneous rate of inhibitory and excitatory postsynaptic currents in the olfactory cortex as well [28]. It has been found that oxytocin depolarizes interneurons and enhances synaptic transmission in the hippocampal area CA1 [27]. Other electrophysiological studies have proved that stimulation of oxytocin receptors reduces spontaneous firing yet enhances excitatory postsynaptic potential onto pyramidal cells in the hippocampus [52].

## 6. The Oxytocin Receptors in the Glial Cells

Glial cells and in particular astrocytes have been identified as targets of oxytocin action. Studies using autoradiography have proved the presence of oxytocin-binding sites located on both soma and processes of astrocyte-like types of cells [61]. Other studies have produced evidence on the effects of oxytocin on astrocytoma cell lines [62, 63], implicating that oxytocin receptors could modulate cellular growth. One study has shown that stimulation of astrocytic oxytocin receptors results in the release of calcium from intracellular stores [64]. The functional aspects of oxytocin receptors were revealed in astrocytes by electrophysiological experiments which proved that oxytocin evokes depolarization of astrocytic membrane potential, and in the context of suckling, it could induce retraction of astrocyte processes [65]. This phenomenon is particularly important to keep in mind in the context of astrocytic participation in neuronal activity. Astroglial-neuronal interactions however are very complex, and in the view of synaptic connectivity, astrocytic transformations are related to the number of synapses. It has been repeatedly demonstrated that the astrocyte oxytocin receptor is coupled to a G protein-coupled receptor and increases intracellular calcium concentration mobilized from IP_3_-sensitive stores [61, 66].

## 7. The Formation and Stability of the Synapse

A recent study has directly shown that oxytocin affects the number of synapses [4]. This study has suggested that the number of excitatory presynapses is increased in cultures from oxytocin receptor knockout mice, whereas the number of inhibitory presynapses is slightly decreased. Moreover, the authors proved that oxytocin exposure of cultured mouse hippocampal glutamatergic neurons caused altered neuronal dendrite complexity and altered numbers of excitatory synapses. Another study found a decreased ratio of GABAergic versus total presynapses in hippocampal neurons cultured from oxytocin receptor knockout mice [67]. In the context of synapse formation, it is important to take into account as well the effects of oxytocin on neurofilaments. It has been demonstrated that oxytocin promotes the formation of filamentous actin (F-actin) networks on the membrane in the cells of the brain cortex [55]. Furthermore, in our study, we have found increased expression of the actin-binding protein drebrin and the intermediate filament vimentin in response to oxytocin [68]. Thus, oxytocin contributes to the regulation of expression of cytoskeletal proteins associated with neurite growth *in vitro*. Taken from a structural point of view, it is important to emphasize that oxytocin receptor knockout mice suffer from reduction of postsynaptic density protein 95 [69]. It implies that oxytocin receptors can be related to the regulation of synapse scaffolding proteins. This conclusion is supported also by our results on the effects of oxytocin on the SHANK family of scaffolding proteins [47]. Higher dendritic complexity has been observed under the effect of oxytocin [70]. Oxytocin has been found to phosphorylate cAMP-responsive element-binding protein (CREB) [30] and other CREB-regulated genes [71]. This could be a regulatory pathway to many scaffolding proteins, cytoskeleton rearrangement, and synapse formation. Oxytocin signaling has developmental dynamics [11], and therefore, it is important to note that early phases of neuronal development are crucial for functionality of mature oxytocin receptor system. Nevertheless, oxytocin receptors have different brain regional localization. A recent study has especially stressed the distribution of oxytocin receptors in the brain cortex [72]. This could be important for cortical organization and the establishment of synapses and neural circuits for complex social behaviors.

## 8. Future Outlook

Even though the mechanisms are far from clear, promising results on the use of oxytocin have been produced by some research groups. One recent study has revealed that early oxytocin treatment could be an effective strategy for the treatment of neurodevelopmental diseases such as Prader-Willi syndrome and autism spectrum disorders [73]. Mutations of the Magel2 gene have been described in patients with autism and a loss of Magel2 is also associated with Prader-Willi syndrome. In general, behavioral and psychiatric disturbances beginning in early childhood are observed in affected individuals [74, 75]. The pathophysiological mechanism is the object of intensive research, and many studies are focusing on the developmental aspects of the oxytocin system. The research presented by Schaller et al. [76] has shown that the hypothalamus of the neonatal Magel2-deficient mouse had significantly lower levels of oxytocin, and oxytocin administration reversed the feeding difficulties in Magel2-deficient murine pups. Furthermore, it has been confirmed that oxytocin plays an important role in the onset of feeding. In mice, Magel2 is highly expressed in postmitotic hypothalamic neurons from early embryogenesis, and it occurs in the highest expression levels in the suprachiasmatic (SCN), the paraventricular (PVN), and supraoptic (SON) nuclei, in particular in vasopressin-positive neurons in the adult brain [77, 78]. It should be emphasized that the developmental effects of oxytocin in the regulation of feeding behavior are different from the studies involving the acute effects of oxytocin on food intake. Oxytocin was found to reduce feeding in animals; nevertheless, its anorexigenic effect depends on the dosage, method of administration, and diet composition [79].

Oxytocin administration in the first postnatal week was sufficient to prevent deficits in social behavior and learning abilities in Magel2-deficient mice. It seems that the impact of oxytocin on the developing brain around the time of birth has long-term consequences on behavior and on cognition. The research revealed that an oxytocin administration in Magel2-deficient mice acts directly on oxytocin-binding sites in brain structures and partially on oxytocin neurons, resulting in the release of endogenous oxytocin and suppressing the accumulation of oxytocin-intermediate forms in oxytocin neurons [76]. The effect of oxytocin on cognition during development has been confirmed in another recent study [80]. These authors have shown that oxytocin influences primate infant cognitive abilities. Moreover, yet another study has clinically proved beneficial effects of continual oxytocin administration on the social symptoms in patients with autism [81]. Nevertheless, the use of oxytocin and its analogs is still under intense debate, and numerous clinical trials of oxytocin in autism spectrum disorders are ongoing [82]. In the context of neurodevelopmental disorders, a therapeutic strategy could include some specific small-size neuropeptides. It seems very likely that one of the factors which plays a role in the pathogenesis of autism is the oxytocin receptor system especially in early development. As mentioned earlier, oxytocin affects the expression of neuron and glial markers in the brain [19], and it appears to be a relevant factor for the regulation of scaffolding proteins as well [47]. It has been suggested that oxytocin could be effective in the early treatment of neurodevelopmental diseases [83]. Development of the oxytocin system is essential for the early postnatal life of mammals; therefore, oxytocin is required in critical windows of time that play a pivotal role in brain development [84]. Indeed, it has been demonstrated that oxytocin receptors are associated with timing of the switch of GABAergic neurotransmission from excitatory to inhibitory during development of the nervous system [85]. Leonzino et al. have shown that the GABA switch is delayed in the absence of oxytocin receptor expression via a mechanism involving a chloride transporter. In this context, it is important to note that it has been recently shown that oxytocin exerts an early and cell-type-specific “priming” effect on developing excitatory neurons [4]. Therefore, it can be concluded that neuronal wiring is influenced by the action of oxytocin. Recent findings could be particularly important for the understanding of pathological processes of neurodevelopmental diseases and their compensation by early treatment.
